# In Vitro Evaluation of Cytotoxic and Pro-Apoptotic Effects of Hesperidin Alone and in Combination with Cisplatin on Human Malignant Melanoma Cell Line (A431)

**DOI:** 10.3390/ph18060854

**Published:** 2025-06-07

**Authors:** Mehmet Uğur Karabat, Mehmet Cudi Tuncer, İlhan Özdemir

**Affiliations:** 1 Department of Histology and Embryology, Medical Faculty, Dicle University, Diyarbakır 21200, Turkey; ugurkrbt@hotmail.com; 2 Department of Anatomy, Faculty of Medicine, Dicle University, Diyarbakır 21200, Turkey; 3 Department of Gynecology and Obstetrics, Faculty of Medicine, Atatürk University, Erzurum 25240, Turkey; ilhanozdemir25@yandex.com

**Keywords:** melanoma, Hesperidin, apoptosis, caspase-3/7, MTT

## Abstract

**Background/Objectives:** Melanoma is an aggressive skin cancer with high metastatic potential and poor prognosis in advanced stages. Hesperidin, a natural flavonoid, has shown anticancer properties across various malignancies. This study aimed to evaluate the antiproliferative and pro-apoptotic effects of Hesperidin, alone and in combination with Cisplatin, on the human epidermoid carcinoma cell line A431. **Materials and Methods:** A431 cells were cultured under standard conditions and treated with different concentrations of Hesperidin and Cisplatin for 48 h. Cell viability was assessed using the MTT assay. Apoptosis was evaluated by Annexin V-FITC/PI staining and caspase-3/7 activity assays. Expression levels of Bax, caspase-3/7, and survivin were measured by RT-qPCR. **Results:** Hesperidin significantly reduced cell viability at both 24 and 48 h. Annexin V/PI staining revealed increased apoptosis, with the highest apoptotic ratio in the Hesperidin + Cisplatin group (*p* < 0.001). Caspase-3/7 activity was markedly elevated in Hesperidin-treated cells. RT-qPCR showed upregulation of Bax and caspase-3/7 and downregulation of survivin. **Conclusions:** Hesperidin demonstrated significant cytotoxic and pro-apoptotic effects in A431 cells. When combined with Cisplatin, a synergistic enhancement of apoptosis was observed. These findings support the potential of Hesperidin as a complementary agent in carcinoma therapy, pending further in vivo and clinical validation.

## 1. Introduction

Melanoma is a malignant tumor arising from melanocytes and represents the most aggressive form of skin cancer. Its global incidence is increasing, and advanced-stage melanoma remains difficult to treat due to high metastatic potential, drug resistance mechanisms, and low response rates [1,2]. Despite advances in targeted and immunotherapies, the median survival in widely metastatic melanoma remains between 6 and 11 months, depending on the site and burden of metastases [3].

Cisplatin is a conventional chemotherapeutic agent that exerts its antitumor effect by inducing DNA damage, leading to cell cycle arrest and apoptosis. However, its clinical use is limited by severe systemic side effects, including nephrotoxicity and chemotherapy-induced resistance [4]. To address these challenges, combining chemotherapeutic agents with natural products has gained increasing attention as a strategy to enhance efficacy and reduce toxicity. 

**Figure 1:**
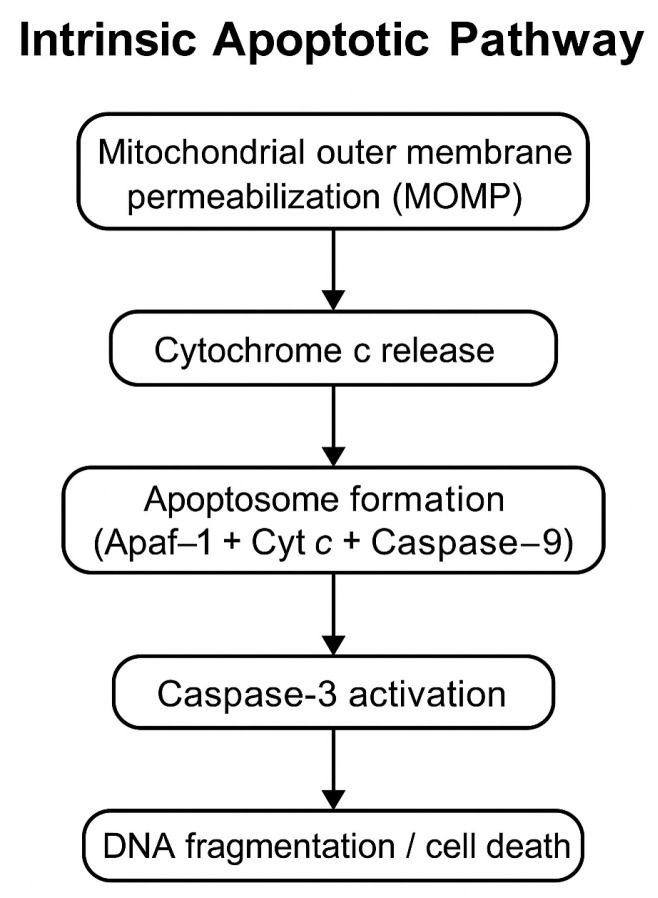
Schematic representation of the intrinsic apoptotic pathway highlighting the role of caspase-3 as a mediator. Mitochondrial damage leads to cytochrome c release, apoptosome assembly (Apaf-1 + cytochrome c + caspase-9), and sequential activation of caspase-3, resulting in apoptotic execution, including DNA fragmentation and cell death.

Flavonoids, a class of plant-derived polyphenolic compounds, are known for their diverse biological activities, including antioxidant, anti-inflammatory, and anticancer effects [5,6]. Hesperidin, a flavanone glycoside predominantly found in citrus peels, has shown antiproliferative and pro-apoptotic effects in malignant cell lines derived from various cancer types, including colon, breast, liver, prostate, and multiple myeloma [7,8]. These effects are often mediated through activation of the intrinsic apoptotic pathway, involving Bax upregulation, caspase-3/7 activation, and downregulation of the anti-apoptotic protein survivin [9,10]. Caspase-3 is a key executioner caspase that acts as a mediator in the intrinsic apoptotic pathway. Its activation reflects not only the presence of apoptosis but also the molecular mechanism by which it is executed (Figure 1).

In addition to its direct anticancer activity, Hesperidin influences several signaling pathways implicated in tumor progression, including PI3K/Akt and NF-κB [11]. Furthermore, its combination with chemotherapeutics like Cisplatin has been shown to augment cytotoxicity while mitigating drug-induced toxicity. For instance, in hepatocellular carcinoma models, Hesperidin co-administration with Cisplatin enhanced treatment efficacy and reduced oxidative damage in liver tissues [12]. Moreover, Hesperidin has demonstrated selective cytotoxicity, preferentially targeting cancer cells while sparing healthy ones, thereby improving the safety profile of chemotherapy [13].

Despite the promising findings regarding Hesperidin’s anticancer effects, studies evaluating its combination with Cisplatin in epidermoid carcinoma or melanoma models remain limited. Given the aggressive and treatment-resistant nature of such cancers, exploring the potential synergy between Hesperidin and Cisplatin is of clinical significance.

In this study, we hypothesize that Hesperidin, alone or in combination with Cisplatin, exerts a synergistic cytotoxic effect in A431 cells via activation of intrinsic apoptotic mechanisms. To test this, we assessed cell viability using MTT assays, evaluated apoptosis using Annexin V/PI staining and caspase-3/7 activity, and analyzed the expression of apoptosis-related genes (Bax, caspase-3/7, survivin) by RT-qPCR. The findings of this study may support the development of Hesperidin as a potential adjunct to chemotherapy for epidermoid carcinoma.

## 2. Results

### 2.1. Cell Viability and Cytotoxicity Analysis (MTT Test)

**Figure 2:**
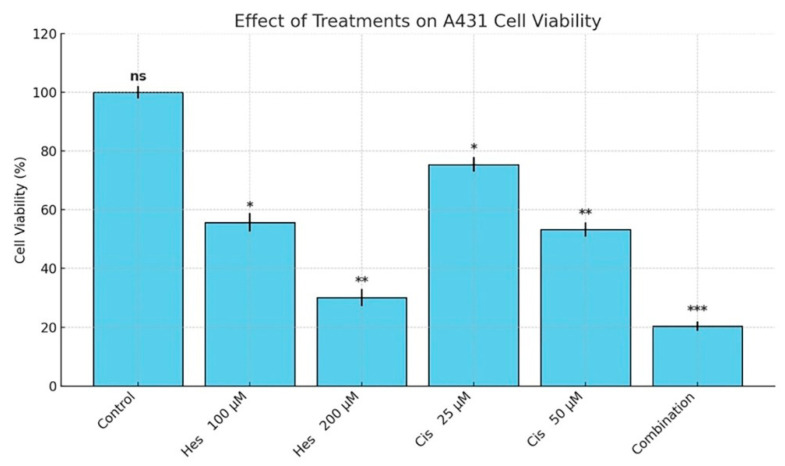
Hesperidin and Cisplatin significantly decreased A431 cell viability after 48 hours of treatment.Statistical significance was determined using one-way ANOVA followed by Tukey’s post hoc test, with comparisons made to the untreated control group. Significance levels are indicated as follows: *p* < 0.05 (*), *p* < 0.01 (**), *p* < 0.001 (***); ns: not significant, Hes: Hesperidin, Cis: Cisplatin.

The cytotoxic effects of Hesperidin and Cisplatin on A431 melanoma cells were evaluated using the MTT assay. Hesperidin treatment significantly reduced cell viability in a dose-dependent manner. After 48 h of incubation, treatment with 100 µM and 200 µM Hesperidin resulted in 45.1% (*p* < 0.05) and 65.2% (*p* < 0.01) reductions in cell viability, respectively. Similarly, Cisplatin also decreased cell viability in a dose-dependent fashion, with 25 µM and 50 µM treatments leading to 37.4% (*p* < 0.05) and 48.4% (*p* < 0.01) reductions, respectively. The combination of Hesperidin and Cisplatin produced a synergistic effect, resulting in a significantly greater reduction in cell viability compared to either agent alone (*p* < 0.001) (Figure 2). Specifically, the combination treatment reduced cell viability to 21.7% ± 2.8, indicating the most pronounced cytotoxic effect among all groups.

Based on the 48 h cytotoxicity data, the half-maximal inhibitory concentrations (IC_50_) were calculated as 108.4 µM for Hesperidin and 32.8 µM for Cisplatin. These IC_50_ concentrations were subsequently used in all downstream experiments.

### 2.2. Caspase-3/7 Activity

**Figure 3:**
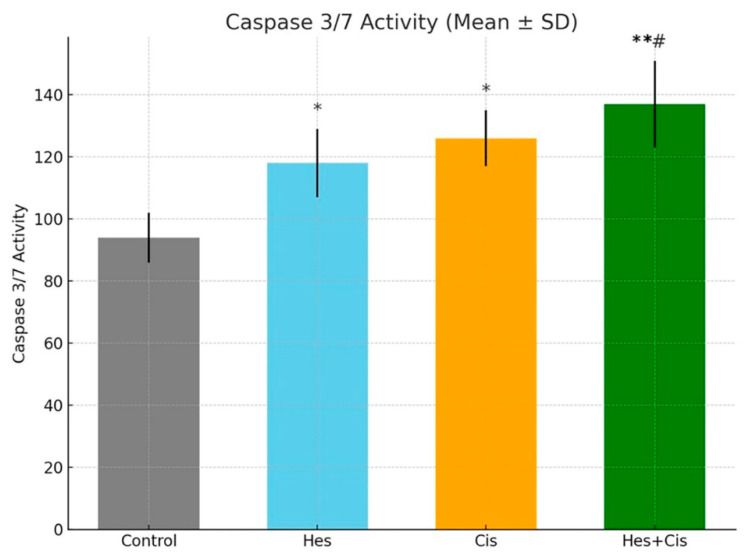
Caspase-3/7 activity levels in A431 melanoma cells following treatment with Hesperidin, Cisplatin, and their combination (Hesperidin + Cisplatin) for 48 h. Cells were seeded in 96-well plates and treated at their respective IC_50_ concentrations (Hesperidin: 108.4 µM; Cisplatin: 32.8 µM). Caspase-3/7 activity was assessed using the Caspase-Glo^®^ 3/7 Assay Kit (Promega), and results are expressed as mean ± standard deviation (SD) from three independent experiments (n = 3). Both Hesperidin and Cisplatin alone significantly increased caspase activity compared to the control group (* *p* < 0.01). The combination treatment resulted in the highest caspase-3/7 activity, which was significantly greater than that of the control (** *p* < 0.001) and also significantly higher than the Cisplatin-only group (# *p* < 0.05). Statistical comparisons were performed using one-way ANOVA followed by Tukey’s post hoc test. Hes: Hesperidin, Cis: Cisplatin.

Caspase-3/7 activity, a key indicator of apoptosis, was assessed to evaluate the pro-apoptotic effects of Hesperidin and Cisplatin. Activation of caspase proteases is a hallmark of apoptotic cell death. In this study, caspase-3/7 activity was measured in cells treated with Hesperidin, Cisplatin, and their combination. Both Hesperidin and Cisplatin alone significantly increased caspase activity compared to the control group (*p* < 0.01). Notably, the combination of Hesperidin and Cisplatin induced the highest level of caspase-3/7 activation, showing a highly significant increase (*p* < 0.001) (Figure 3). Furthermore, caspase activity in the combination group was significantly higher than in either monotherapy group (*p* < 0.01), supporting the presence of a synergistic effect. These findings are consistent with the results obtained from the cell viability assay and Annexin V/PI apoptosis analysis.

### 2.3. Apoptosis Analysis (Annexin V/PI Cell Staining)

**Figure 4:**
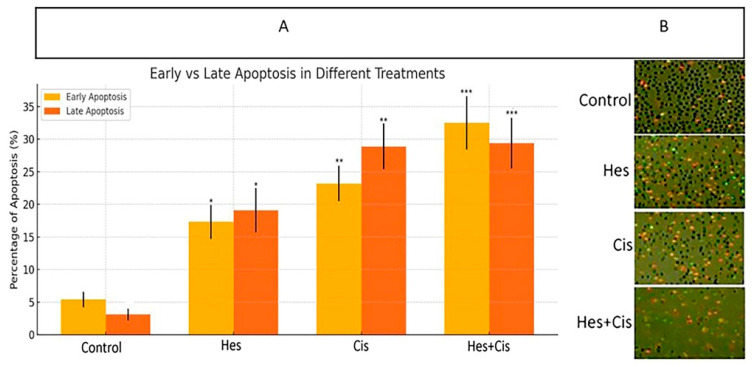
(**A**) Quantification of early and late apoptosis in A431 melanoma cells following 24 h treatment with Hesperidin (108.4 µM), Cisplatin (32.8 µM), and their combination (Hesperidin + Cisplatin). Cells were stained with Annexin V-FITC and propidium iodide (PI), and apoptotic cell populations were analyzed based on fluorescence labeling. Early apoptotic cells (Annexin V^+^/PI^−^, yellow bars) and late apoptotic cells (Annexin V^+^/PI^+^, orange bars) are presented as a percentage of total cells. Both Hesperidin and Cisplatin treatments significantly increased early and late apoptotic cell populations compared to the control group (* *p* < 0.05, ** *p* < 0.01). The combined treatment group (Hesperidin + Cisplatin) exhibited the highest apoptotic rates, with statistically significant increases in both early and late phases compared to all other groups (*** *p* < 0.001). Data are presented as mean ± standard deviation (SD) from three independent experiments. (**B**) Representative fluorescence microscopy images of Annexin V/PI-stained A431 cells under each treatment condition (magnification: ×20, scale bar: 50 μm). Green fluorescence indicates early apoptotic cells (Annexin V-FITC positive), red fluorescence indicates late apoptotic or necrotic cells (PI positive), and unstained cells represent viable (live) cells. The highest intensity and density of apoptotic cells were observed in the combination treatment group. Hes: Hesperidin, Cis: Cisplatin.

The Annexin V-FITC/propidium iodide (PI) double staining method was employed to evaluate whether Hesperidin treatment induced apoptosis in A431 melanoma cells. Hesperidin treatment resulted in a significant, dose-dependent increase in early apoptotic cell populations. Treatment with 100 µM and 200 µM Hesperidin increased early apoptosis rates to 17.3% and 32.5%, respectively, while late apoptosis rates were 19.1% and 29.4%, respectively. Compared to the control group, both early and late apoptotic populations were significantly elevated following Hesperidin treatment (*p* < 0.05). Cisplatin treatment produced a comparable increase in apoptotic cell fractions (*p* < 0.01). Notably, the combination of Hesperidin and Cisplatin led to the most pronounced apoptotic response, with a significant increase in both early and late apoptosis rates compared to all other groups (*p* < 0.001) (Figure 4).

### 2.4. Gene Expression Analysis Findings

**Figure 5:**
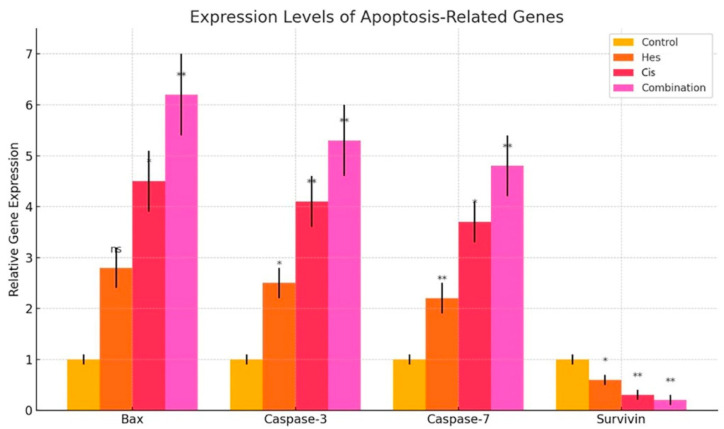
Relative mRNA expression levels of apoptosis-related genes (Bax, caspase-3, caspase-7, and survivin) in A431 melanoma cells following treatment with Hesperidin, Cisplatin, or their combination (Hesperidin + Cisplatin) for 48 h. Gene expression was quantified by RT-qPCR and normalized to GAPDH as an internal control. Expression levels are presented as fold change relative to the untreated control group. Both Hesperidin and Cisplatin alone significantly upregulated Bax, caspase-3, and caspase-7 expression compared to control (*p* < 0.05 or *p* < 0.01), while survivin expression was significantly downregulated. The combination treatment resulted in the most pronounced changes, with Bax, caspase-3, and caspase-7 levels reaching their highest values and survivin expression being nearly abrogated (*p* < 0.01). Data are shown as mean ± standard deviation (SD) from three independent experiments. Statistical analysis was performed using one-way ANOVA followed by Tukey’s post hoc test. Significance indicators: *p* < 0.05 (*), *p* < 0.01 (**), ns: not significant. Hes: Hesperidin, Cis: Cisplatin.

The impact of Hesperidin treatment on the expression of apoptosis-related genes was evaluated by RT-qPCR. The mRNA expression levels of Bax, caspase-3, caspase-7, and survivin were measured. Hesperidin treatment resulted in a significant upregulation of Bax (*p* < 0.01), as well as caspase-3 and caspase-7 (*p* < 0.05), indicating activation of intrinsic apoptotic pathways. Additionally, survivin expression, an anti-apoptotic marker, was significantly downregulated following Hesperidin treatment (*p* < 0.01). Similarly, Cisplatin alone elevated Bax, caspase-3, and caspase-7 expression levels and suppressed survivin expression. Notably, the combination of Hesperidin and Cisplatin induced a more pronounced increase in pro-apoptotic gene expression and a stronger reduction in survivin levels compared to monotherapies (Figure 5).

### 2.5. Hesperidin and Cisplatin Interaction

This combination induced a significant, dose-dependent reduction in cell viability in A431 cells. Results from both the cell viability assay and apoptosis analysis demonstrated a significant increase in the proportion of apoptotic cells in the combination treatment group. This indicates that Hesperidin, when co-administered with Cisplatin, more effectively induces apoptosis in A431 melanoma cells. Bliss independence model analysis further confirmed this interaction, revealing a mild but consistent synergistic effect, particularly at intermediate to high dose levels (Table 1).

### 2.6. Synergy Analysis of Hesperidin and Cisplatin Combination

The pharmacodynamic interaction between Hesperidin and Cisplatin was further explored using multiple synergy evaluation models.

According to the Chou–Talalay model, combination index (CI) values at 50% inhibition (Fa = 0.5) were found to be 0.61, indicating a moderate synergistic effect. CI values at higher fractional effects (Fa = 0.75 and Fa = 0.9) were calculated as 0.58 and 0.52, respectively, demonstrating increasing synergism with elevated drug concentrations.

The Bliss independence model indicated a synergy score of +12.4 at intermediate dose combinations, while the Loewe Additivity model yielded a synergy score of +15.2, further supporting the presence of synergism. Additionally, the Highest Single Agent (HSA) model showed a synergy score of +11.0, particularly at mid-to-high concentration ranges.

Table 2 summarizes the findings from all four synergy models. Overall, these results suggest that the combination of Hesperidin and Cisplatin exhibits a consistent synergistic profile, particularly at moderate and high concentrations, which may enhance therapeutic efficacy while allowing for dose reduction in individual agents.

These comprehensive findings confirm that Hesperidin enhances the cytotoxic effects of Cisplatin in a dose-dependent and synergistic manner across all analytical models applied.

### 2.7. Gene Ontology

**Figure 6:**
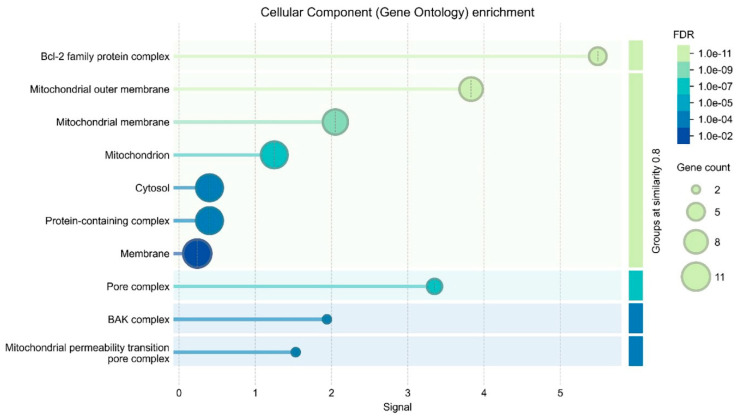
GO enrichment analysis of apoptosis-related genes regulated by Hesperidin and Cisplatin in A431 cells. The bubble plot shows significantly enriched Gene Ontology terms across biological processes and molecular functions. Dot size represents gene count, while color intensity reflects statistical significance (FDR). Key enriched terms include “apoptotic process” (GO:0006915), “cysteine-type endopeptidase activity” (GO:0004197), and “DNA fragmentation” (GO:0006309), indicating activation of intrinsic apoptotic pathways.

In this study, a significant upregulation of caspase-3 gene expression was observed following treatment with Hesperidin alone and in combination with Cisplatin. Caspase-3 is a critical executioner caspase involved in the terminal stages of apoptosis and plays a central role in the orchestration of programmed cell death. According to Gene Ontology (GO) annotations, caspase-3 is associated with key biological processes such as “apoptotic process” (GO:0006915), “DNA fragmentation” (GO:0006309), and “apoptotic signaling pathway” (GO:0097190), all of which are crucial for the regulation of cell death. At the molecular function level, caspase-3 exhibits “cysteine-type endopeptidase activity” (GO:0004197) and “caspase activity” (GO:0097200), enabling it to cleave target proteins at specific aspartate residues. These findings suggest that Hesperidin induces apoptosis by activating intrinsic apoptotic pathways in which caspase-3 plays a key effector role, and this effect is synergistically amplified when co-administered with Cisplatin (Figure 6).

## 3. Discussion

In this study, we demonstrated that Hesperidin exerts significant cytotoxic and pro-apoptotic effects on A431 human melanoma cells and that its combination with Cisplatin results in a synergistic enhancement of these effects. MTT assay results revealed a dose-dependent reduction in cell viability following Hesperidin treatment, with 200 µM causing a 65.2% decrease, while Cisplatin also reduced viability, and their combination yielded the most pronounced inhibitory effect (*p* < 0.001). These findings were supported by Annexin V/PI staining, which showed marked increases in both early and late apoptotic populations, especially in the combination group. Furthermore, caspase-3/7 activity was significantly elevated in all treatment groups, with the highest activity observed in the Hesperidin + Cisplatin group, suggesting enhanced execution of apoptosis. Gene expression analyses via RT-qPCR confirmed the activation of the intrinsic apoptotic pathway, as evidenced by the upregulation of pro-apoptotic Bax, caspase-3, and caspase-7 and the downregulation of anti-apoptotic survivin. The synergy observed in combination therapy was further validated by the Bliss independence model, indicating a mild but consistent synergistic interaction, particularly at moderate to high dose levels. Collectively, these data highlight the potential of Hesperidin to augment Cisplatin-induced cytotoxicity through mitochondrial-mediated apoptosis, suggesting a promising combinatorial strategy for melanoma treatment.

Traditional methods used in cancer treatment can often cause various side effects during the treatment process. Therefore, it is of great importance to develop more targeted, effective, and safe treatment options. In this context, this study, which aimed to evaluate the effects of two different compounds, Hesperidin and Cisplatin, on melanoma, provided important findings for treatment. In this study, the cytotoxic and apoptotic effects of Hesperidin on A431 melanoma cells and its synergistic interactions with Cisplatin were investigated. The findings revealed that Hesperidin dose-dependently decreased cell viability, activated apoptotic pathways, and showed a stronger anticancer effect in combination with Cisplatin.

MTT assay results showed that Hesperidin showed a dose-dependent cytotoxic effect in A431 melanoma cells, and the IC_50_ value was 108.4 µM. This result is consistent with other studies in the literature. For example, Febriansah et al. reported that Hesperidin showed a similar apoptotic effect in MCF-7 breast cancer cells with an IC_50_ value of 120 µM [14]. Here, the 200 µM Hesperidin treatment decreased cell viability by 65.2%, indicating that this flavonoid has a strong cytotoxic potential against melanoma cells. Annexin V/PI staining results revealed that Hesperidin induced apoptotic cell death at both early and late stages. These findings are in agreement with the study by Li et al., which found that Hesperidin induced apoptosis in colon cancer cells [15]. It also overlaps with the mechanism published in 2023, showing that Hesperidin triggers apoptosis by inhibiting the PI3K/Akt pathway [16]. Our RT-qPCR analysis showed that Hesperidin treatment significantly increased pro-apoptotic Bax gene expression (*p* < 0.01) and suppressed anti-apoptotic survivin expression (*p* < 0.01). This suggests that Hesperidin activates the intrinsic (mitochondrial) apoptotic pathway. The increase in Bax may trigger the caspase cascade by disruption of mitochondrial membrane permeability and cytochrome c release [17]. 

Hesperidin, as a flavonoid, promotes the process of apoptosis (programmed cell death) in cancer cells by increasing intracellular reactive oxygen species (ROS) levels. The increase in ROS can lead to cell membrane disruption and cell death. In studies, it has been observed that Hesperidin increases intracellular ROS levels in human melanoma cell lines, and this triggers cell death. This effect of Hesperidin may constitute a strategy for the elimination of treatment-resistant cells in cancer therapy. However, the anticancer effects of Hesperidin are not limited to ROS metabolism; they may also be related to many other mechanisms, such as reducing oxidative stress and controlling inflammation in the cell [18]. Cisplatin is a common chemotherapeutic agent that induces apoptotic cell death through DNA damage. In this study, administration of Cisplatin alone decreased cell viability and increased apoptotic markers as expected. However, the most striking finding was that the combination of Hesperidin and Cisplatin showed a synergistic effect. Bliss analysis confirmed synergy, especially at moderate and high doses. The mechanism underlying this synergistic effect is probably that Hesperidin potentiates the intrinsic apoptotic pathway in addition to the DNA damage caused by Cisplatin. RT-qPCR data showed that the combination treatment increased the expression of Bax, caspase-3, and caspase-7 to a greater extent compared to the treatments alone. This suggests that both components potentiate apoptotic signaling by different mechanisms. Moreover, the effect of Hesperidin in balancing the oxidative stress induced by Cisplatin and repairing cellular damages may have positive results in the treatment process. However, optimization of dosage and treatment protocols is necessary for this combination therapy to achieve clinical success. Hesperidin, due to its antioxidant properties, may play a role in reducing the side effects of Cisplatin treatment, leading to better patient tolerance to the treatment. Hesperidin has also been shown to offer a mechanism of action that prevents cancer cells from becoming desensitized to Cisplatin treatment, making the treatment process more effective [19,20]. The combination of Hesperidin and Cisplatin is a therapeutic strategy that promises to specifically target treatment-resistant melanoma cells. However, further studies are required to confirm the efficacy of this treatment modality at the clinical level. Although the results obtained in preclinical models indicate that this treatment approach is promising, larger and long-term clinical trials are needed to determine the safety and efficacy of both compounds. Furthermore, optimizing the pharmacokinetic properties, dosages, and combinations of both compounds could improve the efficacy of the treatment process. For example, the side effects of Cisplatin treatment can be minimized with Hesperidin, but the dosage needs to be carefully adjusted. The low toxic effect of Hesperidin may help to reduce side effects and improve the treatment process [19]. The apoptotic effects of Cisplatin through DNA damage are described in detail in a recent study published in 2021 [21]. Current strategies for nanoformulation studies published in 2025 may be instructive [22]. This study revealed that Hesperidin exhibited dose-dependent cytotoxic and apoptotic effects in A431 melanoma cells. Importantly, the combination of Hesperidin and Cisplatin exhibited a synergistic effect and markedly increased apoptotic cell death. These findings suggest that Hesperidin is a potential adjuvant agent that can be used in combination with Cisplatin in melanoma treatment. However, these results need to be confirmed by preclinical and clinical studies.

Although the current study demonstrates promising in vitro synergistic cytotoxicity between Hesperidin and Cisplatin, the clinical translation of Hesperidin remains challenged by its limited bioavailability. Human pharmacokinetic studies have shown that the glycosidic form of Hesperidin, particularly Hesperidin -7-O-rutinoside, exhibits poor absorption in the gastrointestinal tract, resulting in low systemic exposure. However, enzymatic conversion to its glucoside form markedly enhances absorption, shifting the uptake site from the colon to the small intestine, as evidenced by a fourfold increase in plasma levels and higher urinary excretion following consumption of α-rhamnosidase-treated orange juice [23]. Furthermore, recent investigations revealed that both the diastereoisomeric composition and micronization process substantially affect Hesperidin’s bioavailability. Specifically, micronized 2S- Hesperidin showed the highest urinary excretion and systemic availability, underscoring the significance of formulation in enhancing therapeutic potential [24]. Finally, literature reviews emphasize that poor aqueous solubility and limited permeability are central to Hesperidin’s low oral bioavailability, and improving these parameters through technological approaches is essential for clinical translation in chronic diseases, including cancer [25]. Collectively, these findings suggest that future studies exploring Hesperidin and Cisplatin combinations should integrate bioavailability-enhancing strategies to ensure efficacy and reproducibility in vivo.

Despite the promising results of this study, several limitations should be acknowledged. First, the in vitro nature of the study restricts the generalizability of the findings to in vivo systems. Although the A431 human melanoma cell line provides a valuable model for evaluating the effects of Hesperidin and Cisplatin, it may not fully recapitulate the biological complexity of primary melanoma tumors in patients. Additionally, although the combination of Hesperidin and Cisplatin demonstrated significant synergistic effects in vitro, their pharmacokinetics, biodistribution, and potential systemic toxicity remain uncharacterized and require further investigation in preclinical animal models and clinical settings. Another limitation lies in the reliance on four synergy models (Chou–Talalay, Bliss, Loewe, and HSA), each with distinct assumptions and sensitivity parameters. While these models are widely employed to assess drug interactions, the variability in synergy scores observed in this study underscores the need for additional validation using alternative computational and experimental synergy frameworks. Furthermore, the long-term effects of Hesperidin and Cisplatin co-administration—particularly concerning the development of drug resistance—were not addressed and should be explored in future longitudinal studies. Importantly, we focused primarily on a limited set of apoptosis-related genes (Bax, caspase-3/7, and survivin). Other apoptotic, anti-apoptotic, or survival-related molecular pathways may also contribute to the observed cytotoxic effects and should be incorporated into future mechanistic investigations. Moreover, the potential impact of cellular dormancy and quiescence must be considered. Since non-dividing tumor cells are often less sensitive to cytotoxic agents like Cisplatin that primarily target proliferating cells, future studies should evaluate the efficacy of Hesperidin and Cisplatin in models enriched with quiescent cell populations. This approach would better reflect the tumor heterogeneity encountered in clinical scenarios. Another key limitation is the absence of non-malignant cell lines in the current study. Evaluating IC_50_ values in both malignant and non-malignant cells is crucial to assess the selectivity and translational safety of the combination treatment. Additionally, analyses of the culture supernatants—such as measuring cytokine levels, ROS generation, or other cellular stress markers—could provide deeper insights into the selective cytotoxic mechanisms of Hesperidin. Lastly, validation of the present findings in additional melanoma and non-melanoma cell lines is warranted to ensure reproducibility and broader applicability across diverse tumor models.

Moreover, previous studies have indicated that Hesperidin exhibits limited pro-apoptotic or antiproliferative activity in certain cancer types. For example, it showed negligible modulation of cell viability and apoptosis in human colorectal cancer cells [26], and it was found to be ineffective both alone and in combination with Cisplatin in ovarian cancer cells [27]. These tumor-specific differences in Hesperidin’s efficacy highlight the need for validation across a broader range of tumor models. Lastly, validation of the present findings in additional melanoma and non-melanoma cell lines is warranted to ensure reproducibility and broader applicability across diverse tumor models.

While this study presents promising in vitro results regarding the synergistic effects of Hesperidin and Cisplatin in human melanoma cells, further research is needed to translate these findings into clinical applications. Future studies should focus on optimizing the dosages and treatment regimens to assess the therapeutic potential and minimize possible side effects in vivo. Additionally, exploring the long-term effects of this combination therapy on melanoma progression and drug resistance is crucial. Investigating the pharmacokinetics and biodistribution of Hesperidin, in combination with Cisplatin, through animal models will be essential for determining its clinical feasibility. Moreover, expanding this research to include a broader range of melanoma cell lines and other cancer types would provide more comprehensive insights into the generalizability of the observed effects. The development of novel delivery systems, such as nanoparticle-based formulations, could further enhance the bioavailability and effectiveness of this combination therapy. Ultimately, large-scale clinical trials will be required to evaluate the safety, efficacy, and potential for personalized treatment strategies based on patient-specific tumor characteristics.

## 4. Material and Methods

### 4.1. Cell Culture

A431 (CRL-1555™) cells, a human epidermoid carcinoma cell line commonly utilized in melanoma-related research due to its aggressive proliferative characteristics, were used in this study. A431 melanoma cells were purchased from the American Type Culture Collection (ATCC, Manassas, VA, USA) and cultured following established guidelines. The cells were maintained in Dulbecco’s Modified Eagle Medium (DMEM; Gibco, Thermo Fisher Scientific, Waltham, MA, USA) enriched with 10% fetal bovine serum (FBS; Gibco) and 1% penicillin–streptomycin solution (Gibco) under standard incubation conditions. Cells were incubated at 37 °C in a humidified atmosphere containing 5% CO_2_. Upon reaching 80–90% confluence, cells were passaged using 0.25% trypsin-EDTA solution (Gibco).

### 4.2. Hesperidin and Cisplatin Administration

Hesperidin (≥95% purity; Sigma-Aldrich, Merck KGaA, Darmstadt, Germany) was dissolved in dimethyl sulfoxide (DMSO) to prepare a 100 mM stock solution and subsequently diluted in DMEM to the desired working concentrations. Cisplatin (Sigma-Aldrich) was dissolved in sterile distilled water to obtain a stock solution at a concentration of 1 mg/mL. A431 cells were seeded in 96-well and 6-well plates and treated with varying concentrations of Hesperidin (100 and 200 µM) and Cisplatin (25 and 50 µM) for 48 h under standard incubation conditions.

### 4.3. Cytotoxicity Analysis (MTT Assay)

The MTT [3-(4,5-dimethylthiazol-2-yl)-2,5-diphenyltetrazolium bromide] assay was employed to determine the viability of A431 cells. Cells were plated in 96-well culture plates at a density of 5 × 10^3^ cells per well. After drug treatment, each well received 10 µL of 5 mg/mL MTT solution and was incubated at 37 °C for 4 h. Following incubation, the supernatant was discarded, and the insoluble formazan crystals were solubilized using DMSO. The optical density was then measured at 570 nm using a BioTek ELx800 microplate spectrophotometer. The results were expressed as a percentage of the absorbance values relative to the untreated control group.

### 4.4. Determination of Effective Dose (IC_50_)

Based on the MTT assay results, the half-maximal inhibitory concentration (IC_50_) values for Hesperidin and Cisplatin were calculated using probit analysis with the SPSS 20.0 statistical software. These values were determined separately for each treatment group and used to identify effective concentrations. The calculated IC_50_ doses were subsequently applied in downstream experiments, including RT-qPCR analysis. Although only two concentrations were shown in Figure 2, additional intermediate concentrations (e.g., 25, 50, 75, 150 µM for Hesperidin and 10, 25, 40 µM for Cisplatin) were tested in preliminary experiments. These values were included in the IC_50_ calculations using probit regression in SPSS 20.0. The resulting dose–response curves are shown in Supplementary Figure S1.

### 4.5. Caspase-3/7 Activity Findings

Caspase-3/7 activity was evaluated using the Caspase-Glo^®^ 3/7 Assay Kit (Promega, Madison, WI, USA) to confirm activation of the apoptotic pathway. Following treatment, cells cultured in 96-well plates were incubated with the caspase reagent according to the manufacturer’s instructions. Plates were then kept at room temperature in the dark for 1 h to allow luminescence development. Luminescence was measured using a microplate reader, and the results were compared to the untreated control group to assess relative caspase activity.

### 4.6. Apoptosis Analysis (Annexin V/PI Cell Staining Method)

Apoptosis rates were evaluated using the Annexin V-FITC/propidium iodide (PI) double staining method. Apoptosis analysis was performed using IC_50_ concentrations: Hesperidin at 108.4 µM and Cisplatin at 32.8 µM, determined from the MTT assay. Following treatment, A431 cells were harvested by trypsinization, washed twice with phosphate-buffered saline (PBS), and resuspended in PBS at a density of 1 × 10⁶ cells/mL. Subsequently, 5 µL of Annexin V-FITC and 5 µL of PI were added to each sample, and the cells were incubated in the dark at room temperature for 15 min. After staining, samples were analyzed under a fluorescence microscope (Olympus BX51; Olympus Corporation, Tokyo, Japan).

Apoptotic status was determined based on fluorescence signals as follows:

- Viable cells were identified as negative for both Annexin V-FITC and propidium iodide (PI) staining.
- Cells in early-stage apoptosis exhibited Annexin V-FITC positivity while remaining negative for PI staining.
- Late-stage apoptotic cells showed positivity for both Annexin V-FITC and PI.
- Necrotic cells were characterized by the absence of Annexin V-FITC staining but were PI-positive.

Cells were examined in at least five random fields per condition, and the number of cells in each category was recorded to determine the distribution of cell populations across experimental groups.

### 4.7. Gene Expression Analysis

Quantitative reverse transcription PCR (RT-qPCR) was employed to evaluate the mRNA expression levels of key apoptosis-associated genes, including Bax, caspase-3, caspase-7, and survivin (BIRC5). Total RNA was isolated using TRIzol™ reagent (Invitrogen, Thermo Fisher Scientific, Waltham, MA, USA) following the protocol provided by the manufacturer. The RNA’s concentration and purity were verified using a NanoDrop™ spectrophotometer (Thermo Fisher Scientific, Waltham, MA, USA). All RT-qPCR assays were carried out using Hesperidin at 108.4 µM and Cisplatin at 32.8 µM, corresponding to the IC_50_ concentrations calculated in cytotoxicity assays.

Complementary DNA (cDNA) synthesis was carried out using the High-Capacity cDNA Reverse Transcription Kit (Applied Biosystems) with 1 µg of total RNA as a template. The synthesized cDNA was then used for RT-qPCR analysis, which was performed on an Applied Biosystems 7500 Real-Time PCR System using SYBR™ Green PCR Master Mix (Thermo Fisher Scientific). The primer sequences of the genes used in the study are as follows (Table 3):

The PCR cycling protocol was performed as follows: Initial denaturation at 95 °C for 10 min, followed by denaturation at 95 °C for 15 s, and elongation at 60 °C for 60 s for 40 cycles. Each sample was analyzed in three technical replicates. Gene expression levels were normalized to GAPDH, which was used as a reference gene. Relative gene expression was calculated using the 2^−ΔΔCt^ method and compared to the control group.

### 4.8. Synergy Evaluation Using Multiple Models

To assess the nature of the pharmacological interaction between Hesperidin and Cisplatin, CI and synergy analyses were performed using four standard models: Chou–Talalay CI method, Bliss independence model, Loewe Additivity model, and HSA model.

For the Chou–Talalay method, the CI values were calculated using CompuSyn software version 1.0 (ComboSyn, Inc., Paramus, NJ, USA) based on the median-effect equation, which determines drug interactions as synergistic (CI < 1), additive (CI = 1), or antagonistic (CI > 1).

Bliss independence and Loewe Additivity analyses were performed with SynergyFinder v2.0 (https://synergyfinder.fimm.fi, accessed on 1 May 2025), a web-based platform that uses dose–response matrices to calculate synergy scores. The Bliss model evaluates the expected non-interacting effect as EBliss = EA + EB – EA × EB, while Loewe’s model is based on the principle that a drug cannot be more effective than itself. Scores above +10 suggest synergism, between −10 and +10 suggest additive effects, and below −10 indicate antagonism.

The HSA model, which compares the combination effect with the most effective single agent, was also used to provide a robust overview of potential interactions. All synergy assays were conducted using 4 × 4 dose matrices and analyzed in triplicates.

### 4.9. Statistical Analysis

All experiments were independently conducted in three replicates, and the outcomes are presented as mean values with their corresponding standard deviations (SD). Statistical evaluations were performed using SPSS software (version 20.0; IBM Corp., Armonk, NY, USA). The Shapiro–Wilk test was applied to examine the normality of the data distributions, while Levene’s test was utilized to verify the equality of variances across groups. Differences among the four experimental groups (Control, Hesperidin, Cisplatin, and Hesperidin + Cisplatin) were evaluated using ANOVA, followed by Tukey’s post hoc test for multiple comparisons. A *p*-value of less than 0.05 was considered statistically significant. Graphical visualizations of the data, including bar charts with error bars, were generated using GraphPad Prism 8.0 (GraphPad Software, San Diego, CA, USA). In this study, drug interactions were interpreted using four standard models: Bliss independence, Loewe Additivity, HSA, and CI. These models differ in their assumptions and definitions of synergy. Bliss Independence assumes probabilistic independence of drug actions; Loewe Additivity is based on the idea that a drug cannot interact with itself and models dose equivalence; the HSA model compares the combination effect with the more effective single agent; and the Chou–Talalay CI model is based on the median-effect principle. In general, synergy is defined as an interaction where the combination effect is greater than the sum of individual effects (CI < 1), additivity is when effects are equal (CI = 1), and antagonism is when the combination is less effective (CI > 1). For this analysis, Combenefit v2.02 software was used [28,29].

## 5. Conclusions

This study provides supportive evidence that the combination of Hesperidin and Cisplatin exerts a synergistic cytotoxic effect in A431 human epidermoid carcinoma cells. The combination enhanced the inhibition of cell viability and promoted apoptosis through mitochondrial-mediated intrinsic pathways. These results suggest that Hesperidin may act as a potential adjuvant to Cisplatin by amplifying its pro-apoptotic effects and possibly reducing its associated toxicity and resistance in vitro.

While these findings are encouraging, they remain preliminary. Further investigations using additional melanoma and non-melanoma cell lines, in vivo models, and comprehensive pharmacokinetic evaluations are required to assess the safety, efficacy, and mechanism of this combination. Future research should also explore optimal dosing strategies, delivery systems, and effects on quiescent or non-malignant cells to determine the translational value of Hesperidin–Cisplatin co-treatment in melanoma and beyond.

## Figures and Tables

**Table 1:** Synergistic effect of Hesperidin and Cisplatin combination in A431 cells according to the Bliss independence model. Hes: Hesperidin, Cis: Cisplatin.

Dose Level	Cis (%)	Hes (%)	Combination (%)	Expected Inhibition (%)	Observed Inhibition (%)	Bliss Difference	Comment
Low	15	30	40	40.5	40	−0.5	Neutral
Middle	25	45	60	58.75	60	+1.25	Mild synergy
High	40	65	80	79	80	+1.0	Mild synergy
Maximum	50	80	90	90	90	0.0	Exact fit

**Table 2:** Synergy evaluation according to CI, Bliss, Loewe, and HSA models.

Dose Level	CI (Chou-Talalay)	Bliss Score	Loewe Score	HSA Score	Interpretation
Low	0.92	+3.1	+4.5	+2.8	Additive
Moderate	0.61	+12.4	+15.2	+11	Synergistic
High	0.52	+14.6	+18.7	+13.3	Strong Synergy

**Table 3:** Primer sequences of the genes.

Gene	Forward Primer (5′-3′)	Reverse Primer (5′-3′)
Bax	TTTGCTTCAGGGTTTCATCCA	ATCCTCTGCAGCTCCATGT
Caspase-3	TGTCATCTCGCTCTGGTACG	AAATGACCCCTTCATCACCA
Caspase-7	CCAGAGCAGAAAGGTGATGG	AGGACACCAGGTCCACAAAT
Survivin	TGCCTGGCAGCCCTTTCTCA	TGCAGTTTCCTCACCTTTCC
GAPDH	GAGTCAACGGATTTGGTCGT	TTGATTTTGGAGGGATCTCG

## Data Availability

All details about the study can be obtained from the corresponding author. The data supporting the findings of this study are not publicly available due to institutional regulations at the center where the study was conducted. Access to the data is restricted and may only be provided by the corresponding author upon reasonable request and with permission from the institution.

## Supplementary Materials

The following supporting information can be downloaded at: https://www.mdpi.com/article/10.3390/ph18060854/s1, Supplementary Figure S1: Dose–response curves of Hesperidin and Cisplatin in A431 cells after 48 hours of treatment. Cell viability was measured using the MTT assay. The dashed horizontal line represents 50% cell viability (IC_50_ threshold).

## Abbreviations

The following abbreviations are used in this manuscript:

RT-qPCR	Reverse transcription-quantitative polymerase chain reaction
IC_50_	Determination of effective dose
cDNA	Complementary DNA
HAS	Highest Single Agent
ROS	Reactive oxygen species
