# Supplementary material for: In Vitro Evaluation of Cytotoxic and Pro-Apoptotic Effects of Hesperidin Alone and in Combination with Cisplatin on Human Malignant Melanoma Cell Line (A431)
Source: Pharmaceuticals (Basel). 2025 Jun 7;18(6):854. doi: 10.3390/ph18060854 (PMC12196430; doi:10.3390/ph18060854)
Supplement: Supplementary file 1 [file pharmaceuticals-18-00854-s001.zip › pharmaceuticals-3672459-supplementary.pdf]

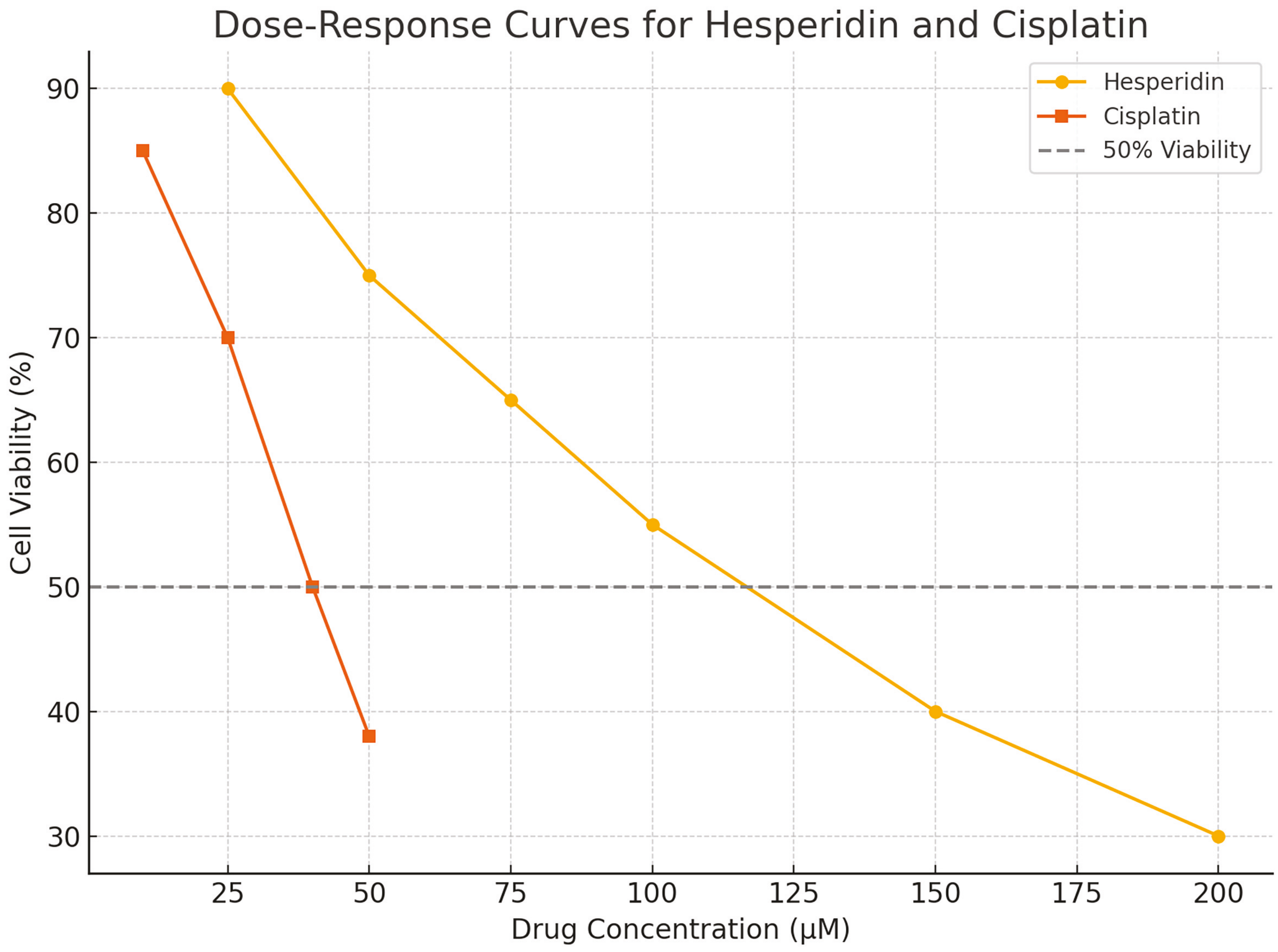


Supplementary Figure S1: Dose-response curves of Hesperidin and Cisplatin in A431 cells after 48 hours of treatment. Cell viability was measured using the MTT assay. The dashed horizontal line represents 50% cell viability (IC50 threshold).
